# Targeted re-sequencing for early diagnosis of genetic causes of childhood epilepsy: the Italian experience from the ‘beyond epilepsy’ project

**DOI:** 10.1186/s13052-020-00860-1

**Published:** 2020-07-06

**Authors:** Elisabetta Amadori, Marcello Scala, Giulia Sofia Cereda, Maria Stella Vari, Francesca Marchese, Veronica Di Pisa, Maria Margherita Mancardi, Thea Giacomini, Laura Siri, Fabiana Vercellino, Domenico Serino, Alessandro Orsini, Alice Bonuccelli, Irene Bagnasco, Amanda Papa, Carlo Minetti, Duccio Maria Cordelli, Pasquale Striano

**Affiliations:** 1 Pediatric Neurology and Muscular Diseases Unit, IRCCS ‘G. Gaslini’ Institute, 16147 Genoa, Italy; 2 Department of Neurosciences, Rehabilitation, Ophthalmology, Genetics, Maternal and Child Health, University of Genoa, Genoa, Italy; 3 Child Neurology and Psychiatry Unit, Department of Medical and Surgical Sciences (DIMEC), S. Orsola Hospital, University of Bologna, Bologna, Italy; 4 Child Neuropsychiatry Unit, Epilepsy Centre, Department of Clinical and Surgical Neurosciences and Rehabilitation, IRCSS ‘G. Gaslini’ Institute, Genoa, Italy; 5 Child Neuropsychiatry Unit, IRCSS ‘G. Gaslini’ Institute, Genoa, Italy; 6 Department of Child Neurology and Psychiatry, Cesare Arrigo Hospital, Alessandria, Italy; 7 Department of Paediatric Neurology, Royal Aberdeen Children’s Hospital, Aberdeen, UK; 8 Child Neurology and Psychiatry Unit, ASL CN1, Cuneo, Italy; 9 Pediatric Neurology, Pediatric Department, Azienda Ospedaliera Universitaria Pisana, University of Pisa, Pisa, Italy; 10 Division of Child Neuropsychiatry, Martini Hospital, via Tofane 71, 10141 Torino, Italy; 11 Department of Child Neuropsychiatry, AOU Maggiore della Carita, Novara, Italy

**Keywords:** CLN2, *TPP1*, Targeted re-sequencing, Next generation sequencing (NGS), Early diagnosis, Epilepsy

## Abstract

**Background:**

Childhood epilepsies are a heterogeneous group of conditions differing in diagnostic criteria, management, and outcome. Late-infantile neuronal ceroid lipofuscinosis type 2 (CLN2) is a neurodegenerative condition caused by biallelic *TPP1* variants. This disorder presents with subtle and relatively non-specific symptoms, mimicking those observed in more common paediatric epilepsies and followed by rapid psychomotor deterioration and drug-resistant epilepsy. A prompt diagnosis is essential to adopt appropriate treatment and disease management strategies.

**Methods:**

This is a prospective, multicentre study on the efficiency of targeted re-sequencing in the early identification of the genetic causes of childhood epilepsy, with particular regard to CLN2. After phenotypic characterization, a 283-gene Next Generation Sequencing panel was performed in 21 Italian children with neurodevelopmental abnormalities, aged between 24 and 60 months, experiencing first unprovoked seizure after 2 years of age.

**Results:**

The average age at enrolment was 39.9 months, with a mean age at seizure onset of 30.9 months and a mean time interval between seizure onset and targeted resequencing of 9 months. Genetic confirmation was achieved in 4 out of 21 patients, with a diagnostic yield of 19%. In one case, the homozygous splice acceptor variant c.509-1G > C in *TPP1* was identified, leading to a CLN2 diagnosis. Three pathogenic variants in *MECP2* were also detected in three patients, including the frameshift variant c.1157_1186delinsA (p.Leu386Hisfs*9) in a girl with negative single gene sequencing. Variants of unknown significance (VUS) were found in 11 out of 21 (52.4%) individuals, whereas no clinically significant variants were observed in the remaining 6 subjects.

**Conclusions:**

Our findings support the efficacy of target re-sequencing in the identification of the genetic causes of childhood epilepsy and suggest that this technique might prove successful in the early detection of CLN2 as well as other neurodevelopmental conditions.

## Background

Epilepsy is one of the most common neurological disorders, affecting approximately 50 million people worldwide (World Health Organization, 2017) with the highest incidence in childhood. Approximately 70/100,000 children younger than 2 years are affected [1]. A definite genetic cause can be identified in 70–80% of cases, including both monogenic and polygenic factors [2]. The genetics of epilepsy is complex and several genetic tests are available [3, 4]. Detailed history taking and accurate phenotypic characterization play a relevant role in the choice of the most appropriate test, as well as in the interpretation of the results [4]. In the era of precision medicine, establishing the genetic aetiology of epileptic disorders is crucial to allow patients to access aetiology-based treatment and management [4].

Late infantile neuronal ceroid lipofuscinosis type 2 (CLN2, OMIM #204500) is an ultra-rare paediatric neurodegenerative disorder with an estimated incidence of less than 0.5 per 100,000 live births [5, 6] and an estimated prevalence of 0.6–0.7 per million in Scandinavia [7]. This condition is caused by biallelic mutations in *TPP1* (OMIM *607998), encoding the lysosomal peptidase named tripeptidyl-peptidase I (TPP1, NM_000391.3) [8–13]. This enzyme catalyses the cleavage of amino-terminal tripeptides from small peptides degraded in lysosomes and exerts a mild endopeptidase activity [13, 14]. TPP1 deficiency causes intra-lysosomal accumulation of autofluorescent storage materials (ceroid lipopigment) and neuronal loss [15]. Children with CLN2 may initially present with subtle and non-specific symptoms, such as speech delay and motor impairment, but invariably develop recurrent seizures and a rapid deterioration of cognitive functions [10–12].

Diagnostic delay is common in CLN2, as early signs are relatively non-specific and mimic those observed in more common paediatric epilepsies. The majority of children are diagnosed around the age of 5 years, [5] after a mean time interval of 22.7 months from the seizure onset [16]. Typically, patients are first assessed by paediatric neurologists or child neuropsychiatrists, less often by general paediatricians, and seizures are among the first clinical signs (BioMarin, personal communication, July 2019). In this phase, genetic testing is very important since early genetic diagnosis of CLN2 would allow to start intrathecal enzyme replacement therapy (ERT) with biosynthetic TPP1 (Cerliponase alfa) before the patient develops the characteristic psychomotor regression. Indeed, this therapy can slow down the cerebral degenerative process responsible for disease progression [17, 18].

We used targeted re-sequencing technology in a selected paediatric epilepsy cohort to investigate the role of early genetic testing to achieve a timely diagnosis of CLN2 and other genetic neurodevelopmental disorders featuring early epilepsy.

## Materials and methods

‘Beyond Epilepsy’ is a project focused on the use of Next Generation Sequencing (NGS)-based techniques for early detection of genetic causes underlying childhood epilepsies. Briefly, Blueprint Genetics and BioMarin offer a no-cost NGS panel, including copy number variants (CNVs) and mitochondrial genome analysis, for diagnostics of genetic causes of paediatric epilepsy. The gene panel includes 416 genes and is available in Europe and the Middle East for children with a first unprovoked seizure [19] (https://blueprintgenetics.com/tests/panels/neurology/beyond-paediatric-epilepsy-panel/).

This project is focused on the evaluation of the utility of genetic testing in epilepsy, in line with the International League Against Epilepsy Genetics Commission [20], as well as the current guidelines on genetic testing in epileptic children provided by the Italian League Against Epilepsy [4].

Seven Italian epilepsy centres from the collaborative network of SINP (Italian Paediatric Neurology Society) enrolled children with epilepsy based on the following inclusion criteria: a) age between 24 and 60 months; b) first unprovoked seizure occurring after 2 years of age; c) language delay or regression and/or motor impairment or regression and/or abnormal electroencephalogram (EEG) and/or abnormal brain magnetic resonance imaging (MRI). Children with known epilepsy aetiology were excluded. Patient data were obtained from clinical records and collected through a questionnaire provided by the referring clinicians.

After obtaining written informed consent for genetic testing from parents or legal guardians, the ‘Beyond Paediatric Epilepsy Panel’ was performed on DNA extracted from peripheral blood. The panel includes 283 genes associated with epilepsies of different aetiology (full list in Additional file: Supplementary Table 1). The identified variants were validated by Sanger sequencing. Variants were interpreted according to population genetics (allele frequency < 0.01 in common frequency databases, such as gnomAD and Exome Aggregation Consortium – ExAC), conservation (Genomic Evolutionary Rate Profiling – GERP score), and predicted impact on protein structure and function through several in silico tools (e.g., Sorting Intolerant From Tolerant – SIFT, PolyPhen, Combined Annotation Dependent Depletion – CADD, Mutation Taster, Human Splice Finder). Candidate variants were further classified according to the American College of Medical Genetics and Genomics (ACMG) guidelines [21].

## Results

**Fig. 1:**
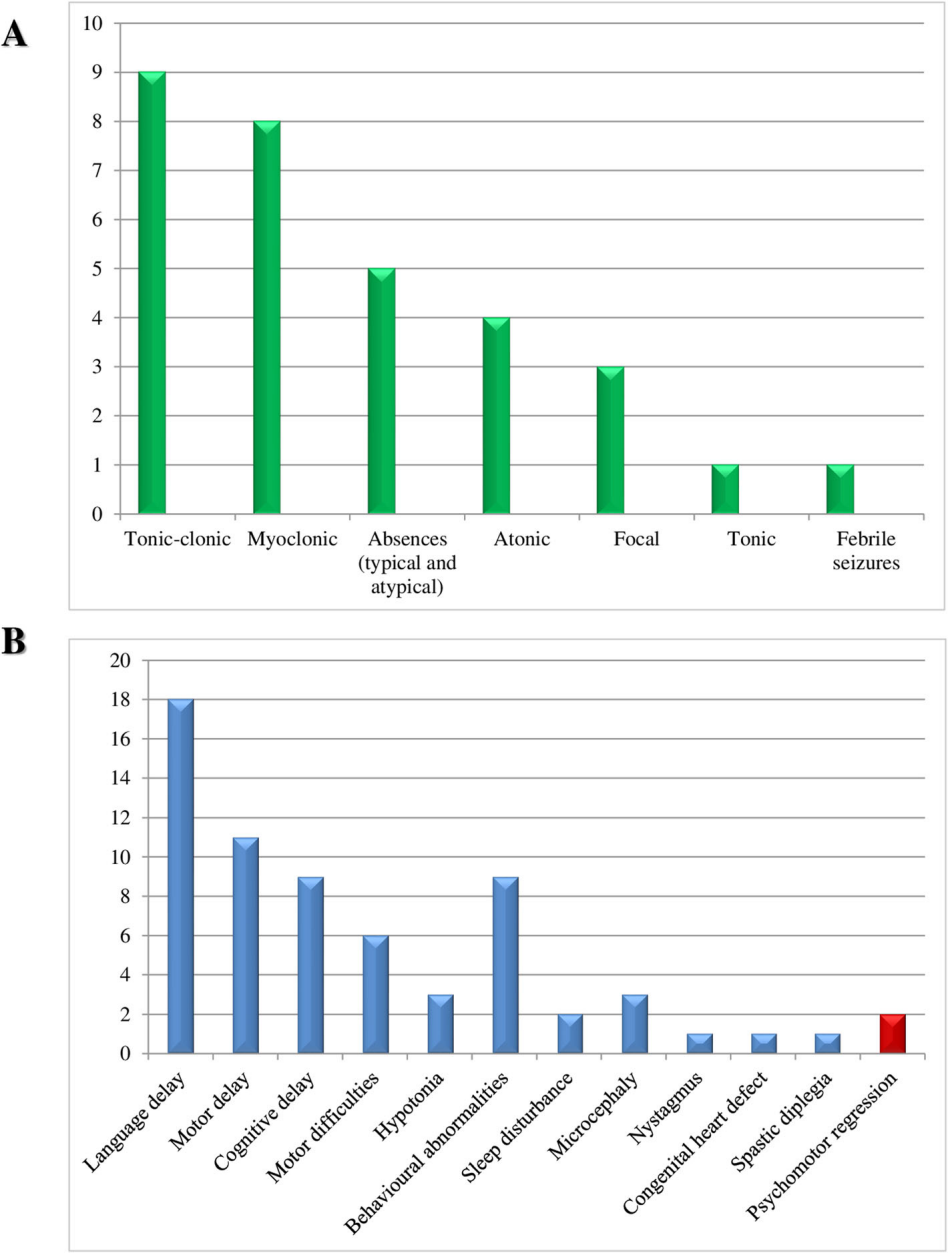
Phenotypic features of this cohort. **a** Seizure types recorded in our cohort. **b** Distribution of the most relevant associated clinical features

Over 3 months, we enrolled 21 patients with different epilepsy phenotypes, of which 8 were males (38.1%) and 13 females (61.9%) (Table 1). The average age at enrolment was 39.9 months (range 57–24 months, median 41 months). Age at seizure onset was 30.9 months on average (range 52–24 months, median 27 months). The average time interval between seizure onset and targeted resequencing was 9 months (range 0–33 months, median 5 months). Patients displayed different seizures types. The most frequent were tonic-clonic seizures, which occurred in 9 out of 21 (42.8%) patients, followed by myoclonic seizures in 8 (38.1%) patients (Fig. 1a). In all subjects, associated neurodevelopmental abnormalities were observed. Language delay was diagnosed in 18 out of 21 patients (85.7%), including 4 nonverbal individuals (22.2%), and motor impairment (clumsiness and ataxia) in 6 patients (28.6%). Other common findings included motor and cognitive delay, behavioural abnormalities (e.g., attention-deficit/hyperactivity and autistic-like behaviour), hypotonia, spastic diplegia, nystagmus, sleep disturbances, congenital heart defect, and microcephaly. Patients #2 and #21 also showed a regression of their developmental skills (Fig. 1b) at 24 months and 45 months of age, respectively (Table 1).

**Table 1:** Clinical features of our cohort

ID/ Sex/ Age (mo)	Consanguinity	Seizure onset (mo)	Seizures semiology	LD	MD	CD	PR (age at onset, mo)	Other clinical features (age at onset, mo)	EEG	Brain MRI	Negative genetic investigations
1/M/52	–	36	TC	+	+	–	–	clumsiness (> 25), hyperactivity and attention deficit (44)	central EA (↑ in sleep)	–	–
2/F/56	–	36	TC; absences	+ nv	+	+ severe	+ (24)	autistic-like behaviour (Rett-like phenotype)	central-temporal EA	–	*MECP2*, *CDKL5, UBE3A*, array-CGH
3/F/24	–	24	Focal to bilateral TC	+	+	+ mild	–	–	frontal and central-temporal EA	–	Karyotype; array-CGH
4/F/29	–	24	TC	+	–	–	–	Clumsiness^b^	diffuse EA sleep dependent	Chiari 1	–
5/M/24	–	24	TC	+	+	–	–	behavioural abnormalities (≥24); sleep disturbances^b^	R frontal-temporal EA	–	–
6/F/50	–	45	Absences; myoclonic	+	+	–	–	–	B temporal EA	–	–
7/M/41	–	24	Myoclonic	+	+	+ moderate	–	mild hypotonia^b^	Generalized EA	–	–
8/M/24	+	24	TC; myoclonic	+	+	+ mild	–	ataxia (≥24)^a^	Generalized EA	–	Karyotype; array-CGH
9/F/50	–	44	TC	–	–	–	–	Hyperactivity and attention deficit (44)	Generalized EA	Chiari 1	–
10/M/54	–	52	TC (previous febrile seizures)	+	–	–	–	–	B posterior EA	–	–
11/F/55	–	27	Focal to bilateral TC; atypical absences	+	–	–	–	hyperactivity (< 27); sleep disturbances (< 27)	L central-parietal EA	–	–
12/F/24	–	24	TC	+ nv	+ Nw	+ severe	–	autistic-like behaviour; microcephaly (Rett-Like phenotype)	Generalized EA	–	–
13/F/31	–	29	TC; myoclonic; atypical absences	+ nv	+ Nw	+ severe	–	hypotonia^b^; autistic-like behaviour; microcephaly^c^ (Rett-like phenotype)	L central-anterior EA	–	Array-CGH
14/F/30	+	24	Tonic	+ nv	+	+ severe	–	hypotonia^b^; autistic-like behaviour (12 mo); nystagmus^d^	B frontal-temporal EA	–	–
15/M/33	–	29	Myoclonic; atonic	–	–	–	–	Hyperactivity (32)	B posterior EA	–	–
16/M/42	–	24	Atonic	+	+	+ mild-moderate	–	Mild spastic diplegia^e^ microcephaly congenital heart defect	Generalized EA	choroid plexus cyst; occipital dysgiria	Array-CGH
17/F/57	–	24	Absences; myoclonic	+	–	–	–	clumsiness (17)	B Parietal EA	WMH	–
18/F/44	–	33	Atonic	+	–	–	–	–	Generalized and focal EA	–	–
19/F/35	–	33	Myoclonic	+	–	–	–	–	Multifocal and generalized EA with PPR^f^	Cerebral atrophy	–
20/M/33	–	24	Focal motor with or without bilateral TC evolution	–	–	+ Moderate	–	–	Focal (independent B temporal-occipital EA)	–	–
21/F/50	–	45	Myoclonic, Atonic	+	–	–	+ (> 45)	tremor (50); ataxia (50)	Multifocal EA	PvWMH Cerebellar atrophy	–

*B* bilateral, *CGH* Comparative Genomic Hybridization, *CD* cognitive delay, *EA* epileptiform abnormalities, *EEG* electroencephalogram, *F* female, *L* left, *LD* language delay, *M* male, *MD* motor delay, *MRI* magnetic resonance imaging, *nv* not verbal, *nw* not walking, *PPR* photoparoxysmal response, *PR* psychomotor regression, *PvWMH* periventricular white matter hyperintensity (T2 weighted image), *R* right, *TC* tonic-clonic, *WMH* white matter hyperintensity

^a^ it began with the acquisition of autonomous walking at 2 years of age; ^b^ these symptoms were reported as always present without an apparent regression; ^c^ Secondary microcephaly from 6 months of age; ^d^ present at the time of the first evaluation (30 months), if earlier; ^e^ evident between 6 months and one year of age; ^f^ EEG with intermittent photic stimulation revealed a PPR at low and medium stimulation frequencies

Electroencephalographic studies revealed several distinct epileptiform abnormalities, including unilateral or bilateral focal anomalies (57.1%), multifocal anomalies (9.5%), and generalized anomalies (33.3%) (Table 1). Intermittent Photic Stimulation (IPS) was performed in all patients, but an epileptiform photoparoxysmal response (PPR) was evoked in patient #19 only. No photosensitivity was registered in the remaining cases. Brain MRI showed nonspecific abnormalities (i.e. choroid plexus cyst, occipital dysgiria, and white matter hyperintensities) in 3 individuals and Chiari I malformation in two patients. Furthermore, patient #19 showed global cerebral atrophy and cerebellar atrophy was observed in the patient #21.

Pathogenic variants were identified in 4 patients, with a diagnostic yield of 19%. The homozygous splicing variant c.509-1G > C in *TPP1* (NM_000391.3) was identified in patient #21. This variant is rare in gnomAD (minor allele frequency – MAF 0.0003997) and was never reported in the homozygous state. It is predicted pathogenic by several in silico tools (Table 2).

**Table 2:** Pathogenic variants identified in this study

ID	Sex	Gene	Genomic position (hg19)	cDNA Change	Protein Change	Effect	Status	GERP	Mutation Taster	SIFT	CADD	Inferred effect (ACMG)
2	F	*MECP2*	X:153296096	NM_004992.3 c.1157_1186delinsA	p.(Leu386Hisfs*9)	Frameshift	HET	5.49	Disease causing	–	–	Likely pathogenic
12	F	*MECP2*	X:153296882	NM_004992.3 c.397C > T	p.(Arg133Cys)	Missense	HET	5.24	Disease causing	Damaging	34	Pathogenic
13	F	*MECP2*	X:153296777	NM_004992.3 c.502C > T	p.(Arg168*)	Stop gained	HET	5.48	Disease causing	Damaging	38	Pathogenic
21	F	*TPP1*	11:6638385	NM_000391.3 c.509-1G > C	–	Altered acceptor site (HSF)	HOM	4.43	Disease causing	–	31	Pathogenic

*ACMG* American College of Medical Genetics and Genomics, *CADD* Combined Annotation Dependent Depletion, *F* female, *GERP* Genomic Evolutionary Rate Profiling, *HET* heterozygous, *HOM* homozygous, *HSF* Human Splice Finder, *SIFT* Sorting Intolerant From Tolerant, *XL* X-linked

According to Human Splice Finder, this *TPP1* variant alters the wild type acceptor splice site, leading to abnormal splicing. It has been previously reported in individuals with classic CLN2 and the phenotype of our patient was consistent with this form of the disease [13]. Besides, the segregation analysis in patient #21 revealed true homozygosity for this variant. Three distinct variants in *MECP2* (OMIM * 300005, NM_004992.3) were further identified. The frameshift c.1157_1186delinsA, p.(Leu386Hisfs*9) variant is absent in gnomAD and predicted disease-causing by Mutation Taster. The two missense variants c.397C > T, p.(Arg133Cys) and c.502C > T, p.(Arg168*) are absent in gnomAD, affect conserved residues (GERP scores of 5.24 and 5.48, respectively) and result in a high CADD score (34 and 38, respectively). All these variants were classified as class IV or V (pathogenic or likely pathogenic) according to ACMG guidelines (Table 2). In Rett syndrome, early truncating variants and large insertions and deletions (INDEL) variants are usually associated with a severe phenotype, whereas missense variants and late truncating variants are associated with milder clinical features [22]. However, phenotype variations are observed in patients carrying the same *MECP2* variant. In our cases (patients #2, #12, and #13) no clear genotype-phenotype correlations could be noticed, as the individuals harbouring the frameshift and stop-gain variants (patients #2 and #13, respectively) displayed a comparable phenotype to the subject carrying the missense *MECP2* variant (patient #12).

Variants of unknown significance (VUS) in 12 different genes (*ALG13*, *SCN9A*, *RELN*, *SYNJ1*, *SPATA5*, *COL4A1*, *PCDH19*, *WDR26*, *CLN3*, *PRODH*, *KIF1A*, *GRIN2B*) were further identified in 11 out of 21 patients (52.4%) (See Additional file: Supplementary Table 2). Eventually, no significant variant was detected in the remaining 28.6% of cases.

## Discussion

We used targeted re-sequencing technology in a selected paediatric epilepsy cohort to investigate the impact of genetic testing in diagnosis of CLN2 and other genetic neurodevelopmental disorders featuring early epilepsy. The ‘Beyond Paediatric Epilepsy Panel’ targets protein-coding exons, exon-intron boundaries (± 20 bps), and selected non-coding and deep intronic variants [19]. This genetic test is particularly useful to detect the genetic cause of neurodevelopmental disorders, as it can detect single nucleotide variants and small INDEL up to 220 bps and CNVs. However, it does not identify balanced translocations, complex inversions, and low-level mosaicism.

We identified the pathogenic splicing variant c.509-1G > C in *TPP1* (NM_000391.3) in a 4-year-old girl with myoclonic epilepsy, language delay, mild cognitive impairment, tremor, ataxia, and neuroimaging abnormalities. Her family history was negative for epilepsy. In three girls (ID #2, #12, and #13), we identified a pathogenic variant in *MECP2*. These subjects presented with a clinical phenotype consistent with Rett syndrome. Noteworthy, previous genetic testing, including karyotype, array-comparative genomic hybridization (CGH), and single-gene sequencing, was performed in 23.8% of the studied subjects (ID #2, #3, #8, #13, and #16) and negative results were obtained in all cases. The frameshift variant c.1157_1186delinsA, p.(Leu386Hisfs*9) in *MECP2* (NM_004992.3) was detected in a subject (ID #2) with a previous negative *MECP2* sequencing test. Indeed, some technical limitations are known to affect the sensitivity of Sanger sequencing (e.g., excessive background noise and errors in the amplification step), especially for the detection of indel variants [23]. These limitations are less relevant in the case of NGS thanks to the multiple independent sequencing reads and the bioinformatics analysis, further supporting the diagnostic power of NGS panels [24].

Using this gene panel, the relative percentage of positive cases in our study is around 19% (4/21). Recently, *Pellacani* et al. performed a systematic review on targeted epilepsy gene panels classified as ‘clinically customized’, ‘commercially available’, and ‘functional network-related’, based on their original design. The diagnostic yield of the panels ranged from 0 to 84.6% [25]. A higher number of positive cases (mean 30.2%) was identified through the ‘Commercial’ panels in comparison to ‘clinically customized’ (mean 26.1%) and ‘functional network-related’ (mean 10.5%) panels [25]. The NGS panel used in our study is a ‘commercially available’ panel with a diagnostic yield (19%) in line with similar panels (range 14–46%) [26, 27]. With specific regard to *MECP2*, a diagnostic rate of around 3.5% has been reported for NGS panels in patients with epilepsy and neurodevelopmental disorders [28]. In our study, the diagnostic rate for *MECP2* was 14%, but this finding might be explained by the selection criteria used and the limited number of patients studied. Similarly, the diagnostic rate for *TPP1* in the general population of children with neurodevelopmental disorders was found to be 1.4% [28], whereas it resulted 4.7% in our study.

NGS techniques are very useful in childhood epilepsy, but the correct interpretation of genetic findings can be challenging. This is particularly relevant for patients with VUS, which may be difficult to interpret despite thorough genetic analysis and phenotypic characterization [4, 29]. We identified several VUS in known disease-causing genes (Supplementary Table 2), which were not consistent with the clinical phenotype and were therefore discarded. The only exception was the c.3269A > G variant in *GRIN2B* (NM_000834.3), but parental segregation revealed that this variant was inherited from the healthy father. No candidate variant was detected in 6 cases, this does not exclude a possible genetic cause of epilepsy in these subjects as NGS panels only include a limited, if numerous, number of genes. Accordingly, the next step will be to perform WES in negative cases.

The early diagnosis of CLN2 is significantly challenging due to the relatively non-specific symptoms at onset [30]. Ideally, the diagnosis should be made as soon as the first seizure occurs. Seizures are the most well-known feature of CLN2, although the epileptic phenotype may be variable. Indeed, while myoclonic seizures and myoclonic jerks are classical findings in CLN2, other seizure types (e.g., febrile, generalized tonic-clonic, absence, and focal with or without secondary generalization) may occur [31, 32]. Other clinical features may be suggestive of CLN2, including language delay (that often precedes the onset of seizures), ataxia, and clumsiness [5, 33]. These symptoms typically suggest the diagnosis in an early disease stage. Indeed, early language delay with the onset of seizures at 2–4 years of age has been recently considered the hallmark of CLN2 disease [32]. Accordingly, a thorough phenotypic characterization is essential in the diagnostic process of children with unprovoked seizures [4].

Electroencephalographic investigations may be particularly helpful in raising the suspicion of CLN2. Indeed, early photosensitivity (PPR at low stimulation frequencies of 1–3 Hz) is a critical pointer of CLN2 [32, 34, 35]. However, PPR may not be present in all CLN2 disease patients and photosensitivity may be missed if EEG is not performed early enough or standardized IPS procedure is not employed [31, 36, 37]. In our study, EEGs with IPS were performed in all patients. The epileptiform PPR was found at low and medium stimulation frequencies in patient #19 only. No photosensitivity was registered in the remaining cases. More specifically, the EEG of patient #21 (the CLN2 disease case) did not show the typical PPR at low frequencies.

**Fig. 2:**
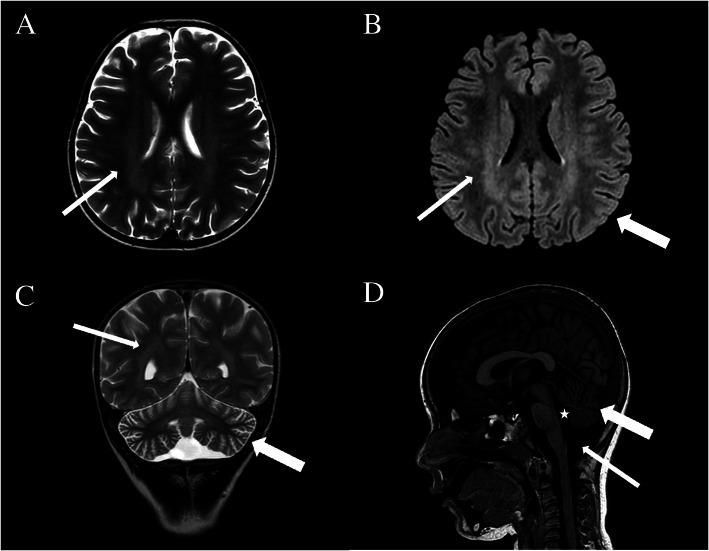
Brain magnetic resonance imaging (MRI) of patient #21. **a** Axial T2-weighted image showing mild hyperintensity in the periventricular deep white matter (thin arrow). **b** Axial fluid attenuated inversion recovery (FLAIR) scan demonstrating hyperintensity in the periventricular white matter, especially in the posterior regions (thin arrow), with preserved myelination in the subcortical white matter (thick arrow). **c** Coronal T2-weighted scan demonstrating mild hyperintensity in the posterior periventricular white matter (thin arrow) and moderate cerebellar atrophy (thick arrow). **d** Sagittal T1-weighted scan showing moderate cerebellar atrophy (thick arrow) with enlarged IV ventricle (star) and cisterna magna (thin arrow). The time from the first seizure to MRI was one month

Another diagnostic clue to CLN2 includes characteristic changes on brain MRI, such as progressive cerebral and cerebellar atrophy and periventricular white matter changes [35]. Accordingly, brain MRI at 46 months of age revealed periventricular white matter hyperintensity and cerebellar atrophy in patient #21 (Fig. 2). Johnson et al., recently showed a gradient from more severely affected posterior structures to mildly affected anterior structures. Moreover, they revealed novel findings which may help to clarify the imaging pattern for the early diagnosis of CLN2 disease, such as subtle changes in both hippocampal architecture and brainstem thinning, particularly involving pontine structures [32]. In this patient, the gene panel allowed a diagnosis of CLN2 within 5 months from the onset of the first seizure. Indeed, either deficient TTP1 enzyme activity or the detection of two pathogenic variants in *CLN2* in trans alone can be diagnostic for CLN2 [15]. After the molecular diagnosis was made, the TPP1 enzyme activity test was also performed and confirmed the enzymatic deficiency, allowing to promptly start the enzymatic replacement therapy (ERT). In this individual, the combined motor and language score on the Hamburg Motor and Language (HML) scale was 4 at the time of diagnosis. In detail, the motor score was 2 (frequent falls, lack of coordination) and language score was 2 (her language skills regressed but her speech remained understandable).

Our study presents some limitations. Firstly, we analysed a cohort of 21 patients, which is too small to be statistically significant for a disease with an estimated incidence of less than 0.5 per 100,000 live births [5, 6]. Second, inclusion criteria were selected to be CLN2-centered but cannot be considered specific for this condition. Nevertheless, we were able to achieve an early diagnosis of Rett syndrome in three individuals, with positive implications on the management of these patients.

## Conclusion

The ‘Beyond Paediatric Epilepsy Panel’ is a powerful diagnostic tool in paediatric patients with epilepsy and neurodevelopmental disorders. Our observations suggest that this NGS-based approach may prove efficient in the early diagnosis of CLN2, allowing the timely adoption of the most accurate treatment strategies (including ERT). These findings also hint to include *TPP1* in the epilepsy NGS panels. We suggest that this panel represents a cost- and time-effective diagnostic weapon in the hands of paediatric neurologists and geneticists, especially when clinical features are not strictly suggestive of a specific disorder.

## Supplementary information

**Additional file 1: Supplementary Table 1.** Full list of the 283 genes included in the ‘Beyond Paediatric Epilepsy Panel’ version used for this study. Abbreviations: MQ, mapping quality score UCSC, University of California Santa Cruz genome browser. **Supplementary Table 2.** Variants of unknown significance identified in this study. Abbreviations: AD, autosomal dominant; AR, autosomal recessive; CADD, Combined Annotation Dependent Depletion; F, female; HEM, hemizygous; HET, heterozygous; M, male; SIFT, Sorting Intolerant From Tolerant; VUS, variants of unknown significance; XL, X-linked.

## Data Availability

The dataset supporting the conclusions of this article are included within the article and its additional file.

## Abbreviations

ACMG: American College of Medical Genetics and Genomics
B: Bilateral
CADD: Combined Annotation Dependent Depletion
CD: Cognitive delay
CGH: Comparative Genomic Hybridization
CLN2: Late-infantile neuronal ceroid lipofuscinosis type 2
CNVs: Copy Number Variants
EA: Epileptiform abnormalities
EEG: Electroencephalogram
ERT: Enzyme replacement therapy
ExAC: Exome Aggregation Consortium
F: Female
FLAIR: Fluid attenuated inversion recovery
GERP: Genomic Evolutionary Rate Profiling
HET: Heterozygous
HML: Hamburg Motor and Language scale
HOM: Homozygous
HSF: Human Splice Finder
INDELs: Small insertions and deletions
IPS: Intermittent Photic Stimulation
L: Left
LD: Language delay
M: Male
MAF: Minor Allele Frequency
MD: Motor delay
MRI: Magnetic Resonance Imaging
NGS: Next Generation Sequencing
nv: Not verbal
nw: Not walking
OMIM: Online Mendelian Inheritance in Man
PPR: Photoparoxysmal response
PR: Psychomotor regression
PvWMH: Periventricular white matter hyperintensity
R: Right
SIFT: Sorting Intolerant From Tolerant
SINP: Italian Paediatric Neurology Society
TC: Tonic-clonic
TPP1: Tripeptidyl-peptidase I
VUS: Variants of Unknown Significance
WES: Whole Exome Sequencing
WMH: White matter hyperintensity
XL: X-linked
