# Supplementary material for: Targeted re-sequencing for early diagnosis of genetic causes of childhood epilepsy: the Italian experience from the ‘beyond epilepsy’ project
Source: Ital J Pediatr. 2020 Jul 6;46:92. doi: 10.1186/s13052-020-00860-1 (PMC7339579; doi:10.1186/s13052-020-00860-1)
Supplement: Supplementary file 1 — Additional file 1: Supplementary Table 1. Full list of the 283 genes included in the ‘Beyond Paediatric Epilepsy Panel’ version used for this study. Abbreviations: MQ, mapping quality score UCSC, University of California Santa Cruz genome browser. Supplementary Table 2. Variants of unknown significance identified in this study. Abbreviations: AD, autosomal dominant; AR, autosomal recessive; CADD, Combined Annotation Dependent Depletion; F, female; HEM, hemizygous; HET, heterozygous; M, male; SIFT, Sorting Intolerant From Tolerant; VUS, variants of unknown significance; XL, X-linked. [file 13052_2020_860_MOESM1_ESM.docx]

**Supplementary Table 1. Full list of the 283 genes included in the ‘Beyond Paediatric Epilepsy Panel’ version used for this study**

| **Genes in the ‘Beyond Paediatric Epilepsy Panel’** |
| --- |
| *ABAT, ABCD1*, ADAR, ADSL, AFG3L2*, AGA, AIFM1, AIMP1, ALDH3A2, ALDH5A1, ALDH7A1, ALG13, AMACR, AMT, AP4B1, AP4E1, AP4M1, AP4S1*, APOPT1, ARG1, ARHGEF9, ARSA, ARX, ASAH1, ASNS*, ASPA, ATP13A2, ATP1A3, ATRX, BRAT1, BTD, CACNA1A, CACNA1H, CACNB4, CASK, CASR, CC2D1A, CDKL5, CERS1, CHD2, CHRNA2, CHRNA4, CHRNB2, CLCN2, CLCN4, CLN3, CLN5, CLN6, CLN8, CNTNAP2, COL4A1, COX15, COX6B1, CPT2, CSF1R, CSTB, CTC1, CTSD, CTSF, CUL4B, CYP27A1, D2HGDH, DARS, DARS2, DCX, DDC, DEPDC5, DHFR*, DNAJC5, DNM1*, DNM1L, DOCK7, DPYD, DPYS, EARS2, ECHS1, ECM1, EEF1A2, EFHC1, EIF2B1, EIF2B2, EIF2B3, EIF2B4, EIF2B5, EPM2A, ETFA, ETFB, ETFDH, ETHE1, FA2H, FAM126A, FAR1*, FARS2, FGF12, FH, FLNA, FOLR1, FOXG1, FOXRED1, GABRA1, GABRB2, GABRB3, GABRG2, GALC, GAMT, GCDH, GCH1, GFAP, GFM1, GJC2, GLB1, GLDC, GLRB, GNAO1, GNB1, GNE, GOSR2*, GPHN, GRIA3, GRIK2, GRIN1, GRIN2A, GRIN2B, GRN, GTPBP3, HACE1, HCN1, HECW2, HEPACAM, HIBCH, HNRNPU, HSD17B10, HSPD1*, HTRA1, HTT, IBA57, IQSEC2, KCNA1, KCNA2, KCNB1, KCNC1, KCNH1, KCNQ2, KCNQ3, KCNT1, KCTD7, KDM5C, KIF1A, L2HGDH, LGI1, LMNB1, LRPPRC, LYRM7#, MAGI2, MARS2, MBD5, MECP2, MED12, MEF2C, MFSD8, MLC1, MOCS1*, MRPL44, MTFMT, MTHFR, MTOR, NACC1, NDUFAF5, NDUFAF6, NDUFS2, NDUFS4, NDUFS7, NDUFS8, NDUFV1, NECAP1*, NEU1, NFU1, NHLRC1, NOTCH3, NRXN1, NUBPL, OFD1, OPHN1, PCDH19, PGK1, PHF6, PIGA*, PIGN*, PIGO, PIGT, PIGV, PLCB1, PLP1, PNKP, PNPO, POLG, POLR3A, POLR3B, PPT1*, PRICKLE1, PRIMA1, PRODH*, PRRT2, PSAP, PTS, PURA, PYCR2#, QDPR, RAB39B, RARS, RELN, RMND1*, RNASEH2A, RNASEH2B, RNASEH2C, RNASET2, RNF216*, ROGDI, SAMHD1, SCARB2, SCN1A, SCN1B, SCN2A, SCN8A, SCN9A, SCO1, SDHAF1, SERAC1, SERPINI1, SIK1, SLC12A5, SLC13A5, SLC19A3, SLC25A1, SLC25A15*, SLC25A22, SLC2A1, SLC35A2, SLC39A8*, SLC46A1, SLC6A1, SLC6A8, SLC9A6, SMS, SNAP25, SNORD118, SOX10, SPATA5, SPTAN1, ST3GAL3, ST3GAL5, STX1B, STXBP1, SUMF1, SUOX, SYN1, SYNGAP1, SYNJ1, SZT2, TAF1, TBC1D24, TBCD, TBCE, TBCK, TBL1XR1*, TCF4, TPP1, TREX1, TSC1, TSC2, TTC19, TUBB4A*, UBA5*, UBE2A, UBE3A*, UNC80, VPS13A, WDR26, WDR45, WWOX, YY1, ZEB2* and ZFYVE26* |

It was carried out sequence analysis and copy number variation analysis of the genes listed.

The average sequencing depth is 143X.

The sensitivity to detect variants may be limited in genes marked with an asterisk (*) or number sign (#).

* Genes with partial, or whole gene, segmental duplications in the human genome are marked with an asterisk (*) if they overlap with an UCSC pseudogene (University of California Santa Cruz genome browser).

# The gene has suboptimal coverage when >90% of the gene’s target nucleotides are not covered at >20x with mapping quality score (MQ>20) reads.

‘Beyond Paediatric Epilepsy Panel’ allows to detect single exon deletion (het or homo) with a sensitivity of 100%, while duplications of 1 to 9 exons (het or homo) can be detected with a sensitivity of 75%.

For more information, see the Blueprint Genetics website [19]

**Supplementary Table 2. Variants of unknown significance identified in this study**

| **ID** | **Sex** | **Gene** | **Position** | **cDNA Change** | **Protein**  **Change** | **Consequence** | **Genotype** | **Polyphen** | **SIFT** | **CADD** | **Inheritance** | **Inferred effect** |
| --- | --- | --- | --- | --- | --- | --- | --- | --- | --- | --- | --- | --- |
| 1 | M | *ALG13* | X:110988087 | NM_001099922.2  c.2887C>T | p.(Pro963Ser) | Missense | HEM | Probably damaging | Tolerated | 25.4 | XL | VUS |
| 3 | F | *SCN9A* | 2:167142962 | NM_002977.3  c.1486G>A | p.(Asp496Asn) | Missense | HET | Benign | Deleterious | 23 | AD, AR | VUS |
| 3 | F | *RELN* | 7:103180840 | NM_005045.3  c.6734C>T | p.(Pro2245Leu) | Missense | HET | Benign | Deleterious | 17.84 | AD, AR | VUS |
| 5 | M | *SYNJ1* | 21:34011333 | NM_003895.  c.3917T>C | p.(Val1306Ala) | Missense | HET | Benign | Tolerated (low confidence) | 14.01 | AR | VUS |
| 6 | F | *SPATA5* | 4:123855338 | NM_145207.2  c.592G>A | p.(Ala198Thr) | Missense | HET | Benign | Tolerated | 13.42 | AR | VUS |
| 7 | M | *COL4A1* | 13:110830248 | NM_001845.4  c.2657C>T | p.(Thr886Ile) | Missense | HET | Probably damaging | Deleterious | 25 | AD | VUS |
| 9 | F | *PCDH19* | X:99596911 | NM_001184880.1  c.2838G>A | p.(Met946Ile) | Missense | HET | Benign | Tolerated | 22.3 | XL | VUS |
| 10 | M | *WDR26* | 1:224599226 | NM_025160.6  c.1061A>T | p.(His354Leu) | Missense | HET | Probably damaging | Tolerated | 33 | AD | VUS |
| 14 | F | *CLN3* | 16:28488944 | NM_001042432.1  c.1210C>T | p.(His404Tyr) | Missense | HET | Probably damaging | Tolerated | 25.7 | AR | VUS |
| 15 | M | *PRODH* | 22:18907088 | NM_016335.4  c.1127G>A | p.(Arg376Gln) | Missense | HET | Probably damaging | Deleterious | 26.5 | AR | VUS |
| 18 | F | *KIF1A* | 2:241685589 | NM_004321.7:  c.2766G>C | p.(Gln922His) | Missense | HET | Probably damaging | Tolerated | 22.6 | AD, AR | VUS |
| 19 | F | *GRIN2B* | 12:13716903 | NM_000834.3: c.3269A>G | p.(Lys1090Arg) | Missense | HET | Possibly damaging | Tolerated | 31 | AD | VUS |

Abbreviations: AD, autosomal dominant; AR, autosomal recessive; CADD, Combined Annotation Dependent Depletion; F, female; HEM, hemizygous; HET, heterozygous; M, male; SIFT, Sorting Intolerant From Tolerant; VUS, variants of unknown significance; XL, X-linked.
